# Development and validation of dependence and craving measures specific to athletes who use anabolic-androgenic steroids

**DOI:** 10.3389/fpsyg.2024.1347211

**Published:** 2024-08-08

**Authors:** Barnaby N. Zoob Carter, Ian D. Boardley

**Affiliations:** School of Sport, Exercise and Rehabilitation Sciences, University of Birmingham, Birmingham, United Kingdom

**Keywords:** anabolic-androgenic steroids, dependence, craving, development, validation

## Abstract

**Background:**

Anabolic-androgenic steroid (AAS) dependence affects approximately 30% of people who use AAS. Presently, measures to assess and diagnose AAS dependence are adapted from scales specific to other forms of drug misuse (e.g., alcohol), containing issues with internal consistency and breadth of construct capture. Additionally, there are no measures available to assess AAS craving, which represents a potentially important coeval factor to AAS dependence. Therefore, this study aimed to develop and provide evidence of validity for measures of AAS dependence and AAS craving.

**Methods:**

Data were collected from male and female strength athletes who use AAS across two samples (*n*_sample 1_ = 206; *n*_sample 2_ = 224). Sample 1 completed the new measures alongside instruments assessing theoretically related constructs (Doping Moral Disengagement, Doping Self-Regulatory Efficacy Scale, craving items from the Wisconsin Smoking Withdrawal Scale, AAS adapted Diagnostic and Statistical Manual for Mental Disorder 4^th^ Edition), whereas Sample 2 completed the new instruments.

**Results:**

Exploratory and Confirmatory Factor Analyses (CFA) with Sample 1 data were used to finalize the item sets for both measures and determine the factorial structures of the AAS Dependence Scale (AASDS) and AAS Craving Scale (AASCS). The AASDS consists of 15-items across five first-order factors that are represented by one second-order factor. The AASCS consists of 16-items across four first-order factors that are represented by one second-order factor. Evidence supporting the concurrent, convergent and discriminant validity of scores obtained with both scales was provided through their associations with the theoretically related variables. CFA with the data from Sample 2 confirmed the factor structures for both scales.

**Conclusion:**

The AASDS and AASCS represent valid and reliable measures of AAS dependence and AAS craving for use in research with strength athletes who use AAS.

## 1 Introduction

Anabolic-androgenic steroids (AAS), primarily used by weightlifters and bodybuilders, are also used by recreational gym goers (Kanayama et al., 2009b). Misuse^1^ of supraphysiological doses^2^ of AAS has been linked with a myriad of undesired health effects (Goldman and Basaria, 2018) including a dependency syndrome, currently estimated to effect up to 30% of people who use AAS (Pope et al., 2014). Although development of substance dependency is thought to be facilitated by drug craving (Donny et al., 2008), craving remains poorly understood (Flannery et al., 2001) and AAS craving has not yet been explored. To explore AAS dependence and craving, valid and reliable assessment instruments are required. The overarching aim of the current research was to develop two such instruments and to validate their scores.

AAS research has demonstrated evidence of a dependency syndrome since late 1980 (Brower et al., 1989). Subsequently, research into this area attempted to diagnose AAS dependence by using diagnostic criteria previously used to identify substance misuse and dependence. The two most accredited criteria for assessing the presence of dependence to substances of misuse are the Diagnostic and Statistical Manual of Mental Disorders criteria (DSM; American Psychiatric Association DSM-5, Task Force, 2013), and the International Classification of Diseases (ICD; World Health Organization, 1992). However, the DSM and ICD disagree on how AAS are viewed. While the ICD volume 10 (ICD-10) does not consider AAS as substances that can cause dependence or symptoms of withdrawal (Midgley et al., 1999), the DSM criteria classifies AAS in the ‘other’ category of substance use disorders. For this reason, research on AAS dependence has primarily utilized the DSM criteria (Kanayama et al., 2009a; Ip et al., 2011; Scarth et al., 2022).

Despite the DSM criteria being primarily used to diagnose dependence toward intoxicating substances of misuse, research identified the presence and prevalence of AAS dependence^3^ among populations of male weightlifters using the DSM criteria (Brower et al., 1991; Kanayama et al., 2009a; Scarth et al., 2022). Unlike commonly misused substances, AAS are not immediately intoxicating (Kanayama et al., 2009a). Instead, AAS are consumed over prolonged periods of time to obtain a delayed reward of increased musculature and strength, rather than administered to obtain the sensation of an instantaneous ‘high’ (Kanayama et al., 2009b). To account for this, researchers have provided recommendations to make the DSM criteria more specific to the use of AAS (Kanayama et al., 2009b).^4^ Amendments included items identifying unsuccessful attempts to stop AAS use due to anxiety over loss of muscle size in drug-free periods, avoidance of important activities in favor of maintaining supraphysiological muscle mass, and excessive time spent training, attending to diet, associating with other AAS users (see Kanayama et al., 2009b). However, these amendments may actually represent risk factors for AAS use, such as muscle dysmorphia, exercise addiction and eating disorders (Cole et al., 2003; Griffiths et al., 2018; Gunnarsson et al., 2022), or even different typologies of AAS users (i.e., ‘Expert’; see Christiansen et al., 2017) rather than dependence alone.

Another method of assessing AAS dependence is the Severity of Dependence Scale (SDS; see Gossop et al., 1995). Initially developed to assess dependence on drugs of abuse (e.g., alcohol, heroin, cocaine and amphetamine), the SDS is a concise and internally consistent dependence measure for such substances (Gossop et al., 1995; Martin et al., 2006). More recently, two studies have reported using an adapted version of the SDS to measure AAS dependence (Cole et al., 2003; Griffiths et al., 2018). Despite this, there are concerns of the use of the adapted SDS to measure AAS dependence. First, like the DSM criteria, the SDS was originally established to identify dependence on intoxicating drugs of abuse (e.g., alcohol, heroin, cocaine, and amphetamine; see Gossop et al., 1995). Second, there is extremely limited evidence for the psychometric properties of the adapted version of the SDS (see Griffiths et al., 2018), as the validity and reliability of scores obtained through its use are largely unknown.

The limitations of the DSM and SDS approaches are further highlighted when one considers current models of AAS dependence. Several models have been presented over the last three decades to try to explain AAS dependence (Brower, 2002; Kanayama et al., 2010; Hildebrandt et al., 2011). In an early model, Brower (1992) indicates AAS may cause dependence via four possible mechanisms: (1) primary reinforcement through neurological reward pathways (e.g., opioid pathways), (2) secondary reinforcement from increased musculature (including increased self-esteem, outside admiration and winning competitions), (3) avoidance of biologically mediated withdrawal symptoms, or (4) avoidance of psychosocial withdrawal symptoms (e.g., depression due to decreased athletic performance). Limitations with this proposed model include difficulties in differentiating between each of the reinforcing factors within survey and case report research (Kean, 2003), as many of these factors often present themselves simultaneously (Yesalis et al., 1990). There also remains a disagreement over the psychoactive nature of AAS. Midgley et al. (1999) indicated AAS dependence is likely to be caused by secondary reinforcing effects due to the personal and socially rewarding nature they exert, rather than via psychoactive stimulation. With the model demonstrating difficulties in practical use within research and the absence of agreement over the psychoactive and reinforcing nature of AAS, use of this model in the development of measures to assess and understand AAS dependence could be problematic.

Bahrke and Yesalis (1994) presented a model of AAS dependence whereby development of dependence originates from socio-cultural contexts, subsequently motivating individuals, primarily males, to engage in an intense and frequent rigmarole of training sessions to build highly muscular physiques. Within this model, it is the training sessions that produce improvements in mood, self-esteem and are associated with controlled dietary programs. Therefore, the reinforcing effects of these anabolic compounds can be attributed to their muscle-building properties (Midgley et al., 1999), and the regimented routines of AAS administration facilitate compulsive training and dietary protocols. Thus, the positively reinforcing effects of this model may relate more to exercise dependence and a desire to boost body capital (Kotzé and Antonopoulos, 2021; Gunnarsson et al., 2022), rather than AAS dependence alone, potentially causing issues for measures of AAS dependence created from this model.

In a later model, Brower (2002) proposed a two-stage process of AAS dependence. The first stage sees AAS being used in high doses to build supraphysiological muscle mass when combined with a strict diet and training regime, reinforced by the development of increased muscle mass this behavior is maintained despite encountering any adverse effects. The second stage is characterized by individuals administering high dosages of AAS activating neurological mediated reward pathways preventing the individual from halting use of AAS (Brower, 2002). The latter stage is characterized by the experience of psychoactive effects (i.e., mood changes, increased aggressive behaviors), and associated with polysubstance use of compounds such as opioids, and can be a target for addiction treatments (Arvary and Pope, 2000; Mhillaj et al., 2015). However, Brower (2002) stipulates there is a dearth of evidence of AAS dependence without associated weight training or ergogenic effects on musculature, therefore the positively reinforcing effects of AAS could be attributed more toward an underlying dependence on improving muscular strength, esthetics, and physical performance.

Kanayama et al. (2010) indicated that AAS dependence may present via three distinct mechanisms: the anabolic effect, the androgenic effect, and the hedonic effect. The anabolic mechanism is modulated by the presence of muscle dysmorphic disorder, whereby an individual will maintain the use of AAS due to a ‘fear’ of losing their musculature when abstaining from AAS administration. The androgenic mechanism points to the effects of hypothalamic pituitary gonadal axis suppression, facilitating the development of AAS induced hypogonadism (ASIH) and associated symptoms (see Tan and Scally, 2009). Therefore, AAS are administered to alleviate the symptoms of ASIH experienced during substance free periods (Brower, 2002). Lastly, the hedonic mechanism demonstrates AAS dependence sharing similarities with dependence to other substances of abuse (e.g., opioids), further demonstrated via animal models (Wood, 2008).

More traditional models based upon the allostatic framework of addiction (Hildebrandt et al., 2011) have been presented, whereby the development of AAS dependence is believed to be both a psychological and a physical construct. Hildebrandt et al. (2011) describes how an individual can improve their hedonic state by implementing protocols of exercise and AAS use simultaneously, thereby improving their hedonic tone and bringing about positive reinforcement through social benefit. Combined with chronic AAS use, psychological dependence is established. Physical dependence is achieved once the individual administers ancillary compounds to negate undesired effects (Hildebrandt et al., 2011). However, evidence in the current literature suggests many individuals who use AAS combine their anabolic compounds with ancillary substances to counteract undesired effects (Kanayama et al., 2010; Pope et al., 2014), therefore this may not be indicative of AAS dependence.

The presence of multiple pathways within models of AAS dependence (Brower, 1992, 2002; Bahrke and Yesalis, 1994; Kanayama et al., 2010; Hildebrandt et al., 2011), demonstrates that AAS dependence is not unidimensional but contains many underlying dimensions, as such it is important that measures are able to identify this by containing multiple factors. Presently, measures used to assess AAS dependence capture it as a single factor (Gossop et al., 1995; Gillespie et al., 2007; Ray et al., 2008) and therefore are limited in representing the likely multidimensional nature of AAS dependence. Research in this area could benefit from a multidimensional measure to discriminate between the underlying dimensions of AAS dependence allowing researchers to identify if specific dimensions are of a greater importance to AAS dependence than others.

To accurately identify, diagnose and further understand AAS dependence, the multidimensional nature identified within existing theories needs to be addressed. Sub-dimensions have been identified within the extant literature, with researchers categorizing AAS dependence as almost unbroken use sustained over time, despite incurring undesired (physical, psychological, and social) effects, and experience of withdrawal symptoms in periods of abstinence (Kanayama et al., 2010). Another sub-dimension identified within models of AAS dependence is the belief that administering chronic supraphysiological doses of AAS improves the effectiveness of an AAS regime in enhancing muscular and strength gains (Brower, 2002). By utilizing Kanayama et al.’s (2010) theory of AAS dependence and multiple sub-dimensions identified within the literature, a more accurate understanding of AAS dependence may be established.

A contributing factor in the development of drug dependency is the presence of drug craving (Drummond, 2001; American Psychiatric Association DSM-5, Task Force, 2013). Believed to manifest itself in a myriad of ways, craving presents as an intrusive and dominating sensation causing an individual substantial distress (Tiffany and Wray, 2012). Craving is recognized as a conscious desire for substance use (Sayette et al., 2000), characterized by a want, urge or compulsion to engage in satiating behavior (Kozlowski and Wilkinson, 1987). With little consensus over the definition of craving, it remains poorly understood (Flannery et al., 2001). Craving is believed to present itself during periods of drug abstinence elicited when experiencing drug-related cues (e.g., environmental cues and drug exposure; Drummond et al., 1995), incentive salience (Berridge and Robinson, 2016), and due to drug expectancy (see Drummond, 2001). Thereby increasing the propensity for drug seeking behavior in individuals with compromised self-efficacy (Marlatt and Gordon, 1985), only satiated by drug use.

Craving research has demonstrated its presence within substance use disorders, including alcohol, tobacco, opioid, cocaine, cannabis, and other psychoactive substances (Serre et al., 2018; Kakko et al., 2019). As such, craving has been included within the ICD-10 diagnostic criteria for substance dependence (World Health Organization, 1992), and more recently in DSM-V (American Psychiatric Association DSM-5, Task Force, 2013) for substance use disorder. However, despite consensus that drug craving has a role in drug dependency (Tiffany and Wray, 2012), there remains a dispute among researchers on the presence of a relationship between craving and relapse, indicative of a substance dependency syndrome (Wray et al., 2013). Furthermore, craving has been established as a state-like construct due to daily fluctuations in response to internal and external stimuli (Sayette et al., 2000; Serre et al., 2015), whereas dependence is considered a more stable construct (Geiser et al., 2017; Flannery et al., 2019). Therefore, to identify the presence of craving alongside dependence, it may be more beneficial to retain craving as an independent measure rather than consider it a symptom of dependence and incorporate it into measures of substance dependence.

Research on craving has predominantly focused on alcohol and smoking, subsequently seeing the development of many measures suited to these substances (Sayette et al., 2000), and their adaptation to suit research of other substances of misuse (Tiffany et al., 1993). With no widely accepted or drug specific standardized instruments available, the assessment of drug craving has been diverse (Tiffany and Wray, 2012). This leaves researchers in a predicament where they must pick the most suited measure available, leading to inconsistencies when attempting to identify an appropriate measure (Sayette et al., 2000). Despite the development and validation of multi-item craving measures (Raabe et al., 2005) identifying the varied nature of drug craving, single-item scales remain the most commonly used within craving research (Tiffany and Wray, 2012). This somewhat limits our understanding of drug craving as unidimensional single-item measures are unable to reflect the different multifaceted theories of drug craving (Tiffany, 1990; Tiffany et al., 2000).

Extant research looking at the mechanisms of AAS dependence has identified behaviors associated with craving through animal models. Conditioned place preference and drug seeking behaviors, synonymous with drug craving, have been identified through environmental cues associated with AAS (Wood, 2008). Furthermore, medications to alleviate the symptoms of craving (e.g., Naltrexone) have been seen to inhibit behaviors of self-administration of AAS among animal models (Wood, 2008), supporting the notion AAS can induce a craving-like response. However, some researchers believe animal models are limited in explaining the nature of craving (Mezinkis et al., 2001) due to the inability to communicate sensations and perceptions associated with drug use (Drummond, 2001). Thus, with literature suggesting the presence of AAS craving it is important to further explore AAS craving to better understand whether it associates with AAS dependence.

To accurately identify and diagnose AAS craving and any existing sub-dimensions, a new measure should aim to represent the multi-dimensional nature of the construct (Tiffany and Wray, 2012). Researchers have characterized craving as eliciting several experiences upon an individual, these include cue-elicited craving (Drummond et al., 1995), outcome expectancy (Marlatt and Gordon, 1985), and associated positive and negative mood states (Tiffany et al., 2000; Shiffman and Waters, 2004). Cue-elicited craving has been identified within alcohol research, whereby an individual associates their use of a substance (e.g., alcohol) with an environment (e.g., a bar), and elicits a desire or urge to administer the substance if immediate substance use does not take place (Anton, 1999). Outcome expectancy is explained by the motivation and desire to administer a substance due to the positive outcome of its use, increasing the likelihood of drug-seeking behaviors (Marlatt and Gordon, 1985). The effects of mood on craving have been identified within smoking research, whereby negative affect will increase craving and increase the risk of substance use in periods of abstinence (Shiffman and Waters, 2004). While for positive affect craving is facilitated by pleasurable and positively reinforcing effects increasing the risk for drug seeking behavior (Baker et al., 1986). By utilizing Niaura’s (2000) theory of drug craving and multiple sub-dimensions identified within the literature exploration of AAS craving may be conducted.

Therefore, the overall aim of this study was twofold. First, we aimed to develop measures of AAS dependence and AAS craving and validate their scores. As part of this, we aimed to determine the number of sub-dimensions within each construct to understand the components of AAS dependence and craving and the level of these sub-dimensions within each measure. Based on the theories of dependence and craving previously discussed, we hypothesized: *H1a)* AAS dependence would have a five-factor structure covering major aspects of AAS dependence (e.g., Increase use of AAS due to dissatisfaction with the effectiveness of current AAS regime, AAS use to self-medicate withdrawal-like symptoms, and continued use despite the experience of adverse physical, psychological and socio-occupational effects attributed to AAS use) and *H2a*) AAS craving would present with a 4-factor structure, reflecting the various dimensions of AAS-associated craving (e.g., drug expectancy, environmental cues, and positive and negative mood states). Second, we aimed to determine the presence of a higher order factor for both scales, which would support the existence of general dimensions of AAS dependence and AAS craving. Based upon our previous arguments, we hypothesized: *H1b)* the AAS dependence measure would demonstrate a higher order factor, supporting the existence of an overarching concept of AAS dependence and *H2b*) the AAS craving measure would demonstrate a higher order factor, supporting the existence of an overarching concept of AAS craving. To summarize, this research sought to develop two psychometric instruments: an AAS dependence scale and an AAS craving scale. Throughout the study we followed the guidelines and procedures for instrument development and validation present within the research literature (i.e., Messick, 1995; Fabrigar et al., 1999; Clark and Watson, 2019).

## 2 Methods

Throughout this study we considered five of the six aspects of construct validity proposed by Messick (1995); content, structural, substantive, generalizability, and external. Expert opinion was used for the content aspect via identifying the representativeness and quality of the items for each of the newly developed measures. The structural aspect identifies if the scoring structure is in alignment with the structure of the domains being assessed, this was achieved through factor analysis. The substantive aspect of construct validity was addressed within the study, by examining the association of the scores from our new measures and those from theoretically associated variables. To identify the extent of score properties and interpretations generalizing to and across groups and settings, multisample analysis was conducted. The presence of convergent and discriminant validity was addressed though the external aspect, this was achieved through association with theoretically relevant instrument scores. The final component of construct validity, consequential, is identified through positive and negative consequences occurring from the use of the new measures. As such, this was beyond the scope of an instrument development and validation study, and more applicable to future applications of the measures created within the study.

### 2.1 Item development

By reviewing existing measures assessing the constructs of interest and the current literature, we developed two pools of items designed to capture the various aspects of AAS dependence and craving (Clark and Watson, 2019). In accordance with Clark and Watson (2019), items were selected to cover the major content of AAS dependence (Brower, 2002; Kanayama et al., 2010) and craving (see Marlatt and Gordon, 1985; Drummond et al., 1995; Tiffany et al., 2000; Shiffman and Waters, 2004). Twenty-three items representing AAS dependence and 27 items for AAS craving were generated. Items were either adapted from those used in existing scales (i.e., DSM-IV, ICD-10, Wisconsin Smoking Withdrawal Scale, and The Alcohol Craving Questionnaire) to make them relevant to AAS (*n* = 12 for AAS dependence and *n* = 15 for AAS craving; World Health Organization, 1992; Welsch et al., 1999; Raabe et al., 2005; Kanayama et al., 2009) or created based upon relevant theory^5^ (*n* = 11 for AAS dependence and *n* = 12 for AAS craving). Both the AAS dependence and craving items were provided with a response format of a 7-point Likert scale anchored by 1 (strongly disagree) and 7 (strongly agree), based upon extant guidelines in the literature (see Clark and Watson, 2019). This response format was used during expert panel analysis and all subsequent data collections. This format has been noted within the literature to present the best compromise between reliability, validity, discriminatory power, and respondent preference (Preston and Colman, 2000).

Item pools were subjected to content validity assessment to establish whether they represented the phenomenon they intended to measure (Dunn et al., 1999; Kline, 2005). The most effective way to evaluate content validity is via expert opinion. Following the guidelines of Dunn et al. (1999), items were sent to 22^6^ academics and healthcare workers with cogent experience who had not been involved in item development. Each expert had a PhD in sport psychology, psychology, neuroscience, a medical degree, or were employed within the healthcare sector or as a harm reduction worker with experience of AAS. To establish evidence for the content validity of the items, we presented the item set to our expert panel in a survey consisting of four sections: (i) definition of dependence and content validity assessment for the dependence items, (ii) assessment of format and response items for the dependence items, (iii) definition of craving and content validity assessment for the craving items, (iv) assessment of format and response items for the craving items. Within Sections i and iii, the relevant definition was presented followed by the pertinent items. The experts were then asked to evaluate (a) how representative each item was of the definition on a 7-point Likert scale anchored at −3 (*not at all representative*) and 3 (*very representative*), and (b) comment on each of the item’s relevance to the definition.

Mean expert ratings were computed for each item, following guidelines in the literature surrounding item development (see Hambleton, 1980); any rating that deviated considerably from the other expert scores was removed. Deviant scores were defined as those that equated to or exceeded two response options lower than the next score (e.g., scored at −2 when the next lowest item was 0). Items with a mean expert rating of 1.0 or more were retained, while items scored at 1.0 or less were revised based upon expert panel comments. Out of the 23 items for the AAS dependence scale 11 underwent minor alterations and the remaining 12 remained unchanged. Out of the 27 items for the AAS craving scale, 11 saw minor amendments and the remaining 16 went unchanged. Ten members of the original expert panel examined content validity of the revised items, alongside colleagues from our research group not involved in item creation. Feedback on amended item wording was positive, with only minor adjustments required. Following these stages of item development, the 23 dependence items and 27 craving items were taken forward to the main construct validity phase of the study. Sample size was in accordance with relevant guidelines (Bryant and Yarnold, 1995; Lounsbury et al., 2006; Kline, 2015) who indicate that five participants per item and/or a sample larger than 200 participants provides sufficient power.

### 2.2 Participants

#### 2.2.1 Sample 1

Participants (*N* = 206) originated from 31 countries (*n*_USA_ = 41.7%; *n*_UK_ = 26.2%; *n*_Canada_ = 10.7%, *n_Other_* = 21.4%; see Table 1), and the majority reported being male (90.3%) and heterosexual (85.0%). Participant average age was 32.04 years (*SD* = 9.5), marital status was reported as being single (35.9%), in a relationship (34.5%), married (28.1%) or divorced (1.5%). Employment status indicated participants as unemployed (1.5%), on temporary benefits (2.4%), students (13.6%), on a pension (1.0%), dependent on others (1.0%), part-time employed (8.7%), full-time employed (65.5%), self-employed (3.9%), or other (2.4%). Participants self-reported their age at AAS initiation (*M* = 25.4 years, *SD* = 6.5), the total number of cycles they had run up to the data collection (*M* = 10.3, *SD* = 19.3), number of cycles they had run in the past 12-months (*M* = 1.8, *SD* = 1.2), and total number of years they have been using AAS (*M* = 5.7, *SD* = 6.7).

**Table 1:** Descriptive statistics describing Sample 1 (*N* = 206) and Sample 2 (*N* = 224) demographics and history of AAS use.

	Sample 1			Sample 2		
	Percentage	Mean	SD	Percentage	Mean	SD
Nationality						
United Kingdom	41.7%			78.1%		
United States	26.2%			12.9%		
Canada	10.7%			1.3%		
Other	21.4%			7.7%		
Gender						
Male	90.3%			96.4%		
Female	5.3%			3.6%		
Prefer not to say	4.4%			0.0%		
Sexual orientation						
Heterosexual	85.0%			-		
LGBTQ+	15.0%			-		
Age		32.04	9.50		42.47	10.80
Marital Status						
Single	35.9%			-		
In a relationship	34.5%			-		
Married	28.1%			-		
Divorced	1.5%			-		
Employment status						
Unemployed	1.5%			1.8%		
Temporary benefits	2.4%			1.8%		
Student	13.6%			8.4%		
Pension	1.0%			0.4%		
Dependent on other	1.0%			1.0%		
Part-time employed	8.7%			4.0%		
Full-time employed	65.5%			67.4%		
Self-employed	3.9%			15.2%		
Other	2.4%					
Age of AAS Initiation		25.40	6.50		32.48	10.79
Number of Cycles Run		1.80	1.20		2.14	1.45
Years of AAS Use		5.70	6.70		15.56	11.02

AAS, Anabolic-Androgenic Steroids; SD, Standard Deviation; LGBTQ+, Lesbian, Gay, Bisexual, Transgender, Queer, Intersex, Asexual, and Others. Sexual Orientation and Marital Status data was only collated for Sample 1.

#### 2.2.2 Sample 2

Participants (*N* = 224) originated from 17 countries (*n*_UK_ = 78.1%; *n*_USA_ = 12.9%; *n*_Canada_ = 1.3%. *n_Other_* = 7.7%; see Table 1). Participants were male (96.4%), average age was 42.47 years (*SD* = 10.76). Employment status was unemployed (1.8%), on temporary benefits (1.8%), students (8.4%), on a pension (0.4%), dependent on others (1.0%), part-time employed (4.0%), full-time employed (67.4%), or self-employed (15.2%). Participants self-reported their age at AAS initiation (*M* = 32.48 years, *SD* = 10.79), the number of cycles they had run in the last 12-months (*M* = 2.14, *SD* = 1.45), and the number of years they have been using AAS (*M* = 15.56, *SD* = 11.02).

### 2.3 Measures

#### 2.3.1 Factorial, convergent, and discriminant validity, and internal consistency

To establish evidence for construct validity, we sought evidence for factorial, convergent and discriminant validity via scores from the new measures. Alongside this, we tested for internal consistency. To establish evidence for factorial validity a two-stage approach proposed by Fabrigar et al. (1999) was implemented, whereby Sample 1 identified the dimensions represented in a measure, while Sample 2 confirmed the number and nature of the identified dimensions. Convergent validity was established with Sample 1,^7^ by associating scores of the new measures with pre-existing measures identifying the same constructs (Kline, 2005), in this case the measure for AAS dependence and AAS craving were compared to the AAS amended DSM criteria (Kanayama et al., 2009b) and the AAS adapted Wisconsin Smoking Withdrawal Scale (AAS-WSWS; Welsch et al., 1999), respectively. Discriminant validity was established by comparing the intercorrelations among the subscales of each respective measure. Effect sizes (i.e., small [0.10], medium [0.30] and large [0.50]) for correlation coefficients were determined in accordance with Cohen (1992). Internal consistency was assessed using Cronbach’s alpha utilizing cut off values predetermined within the present literature (see Nunnally and Bernstein, 1994) of unacceptable (*α* < 0.5), poor (*α* ≥ 0.5 to 0.6), questionable (*α* ≥ 0.6 to 0.7), acceptable (*α* ≥ 0.7 to 0.8), good (*α* ≥ 0.8 to 0.9), and excellent (*α* ≥ 0.9).

#### 2.3.2 AAS dependence

The nine-item AAS adapted DSM criteria (Kanayama et al., 2009b) was used to measure AAS dependence. Participants read statements regarding their use and effects associated with their use of AAS (e.g., “over the last 12 months, have you increased the dose/s of steroid/s you are using due to being dissatisfied with your previous results?”) and indicated their level of agreement on a 7-point Likert scale, anchored at 1 (*strongly disagree*) to 7 (*strongly agree*). This scale demonstrated good internal consistency (*α* = 0.82).

#### 2.3.3 Craving

The four-items of craving from the Wisconsin smoking withdrawal scale (Welsch et al., 1999) were adapted to suit AAS craving in this study (AAS-WSWS). The craving items looked at the impact, frequency, and thoughts about the use of AAS on the day to day lives of participants (e.g., “I have trouble getting steroids off my mind”). Participants were instructed to respond to these items using a 7-point Likert scale anchored at 1 (*strongly disagree*) and 7 (*strongly agree*). The scale demonstrated excellent internal consistency (*α* = 0.92).

#### 2.3.4 Moral disengagement

The doping moral disengagement scale (DMDS; Boardley et al., 2018) was used to measure doping moral disengagement. Moral disengagement has been previously associated with AAS use and craving (Boardley et al., 2018; Ahmadi et al., 2019), therefore the DMDS was used within this study to establish evidence for the concurrent validity of scores obtained with the new measures of AAS dependence and craving. The DMDS includes 18-items examining various mechanisms through which people can justify and rationalize doping (e.g., “Compared to most lifestyles in the general public, doping is not that bad”). Participants were instructed to indicate their level of agreement with each statement using a 7-point Likert scale anchored at 1 (*strongly disagree*) and 7 (*strongly agree*). The DMDS has demonstrated good levels of reliability (i.e., test–retest) and validity (i.e., factorial, convergent and discriminant validity), and excellent internal consistency (0.95 and 0.96; see Boardley et al., 2018). The scale within this study demonstrated good internal consistency (*α* = 0.88).

#### 2.3.5 Self-regulatory efficacy

The doping self-regulatory efficacy scale (DSRES; Boardley et al., 2018) was employed to assess doping self-regulatory efficacy. Self-regulatory efficacy has previously been associated with use of AAS and drug craving behaviors (Shadel and Cervone, 2006; Boardley et al., 2018), and was therefore used to establish evidence for the concurrent validity of scores obtained with the new AAS dependence and craving measures. The DSRES includes six items examining the strength of people’s belief in their ability to resist internal and external pressures to dope (e.g., “How confident are you in your ability to ignore the temptation to dope when feeling down physically?”). Participants were instructed to read the six statements and indicate their level of confidence using a Likert scale anchored at 1 (*no confidence*) and 5 (*complete confidence*). The DSRES been shown to have good levels of reliability (i.e., test–retest) and validity (i.e., factorial, convergent and discriminant validity), and excellent internal consistency (0.93 and 0.94; see Boardley et al., 2018). The scale within this study demonstrated good internal consistency (*α* = 0.88).

#### 2.3.6 Use of AAS

Patterns of use for AAS were also assessed. Status of use was determined by items enquiring if participants were presently ‘on-cycle’, ‘off-cycle’, ‘blasting’, ‘cruising’, or on ‘testosterone replacement therapy (TRT)’. Weekly dose of AAS were self-reported (i.e., “Please indicate what estimated combined dosage of anabolic steroid/s you are currently using”). Response options ranged from ‘Nothing (i.e., off-cycle)’ to ‘Over 2 g per week’. Ranges of AAS doses were based upon literature on therapeutic doses (Quaglio et al., 2009), findings from a recent literature review (Pope et al., 2014), and primary research papers (Parkinson and Evans, 2006; Yu et al., 2014), indicating current understanding of low (i.e., clinical doses <300 mg per week), medium, and high doses (i.e., > 1,000 mg per week) of AAS. Participants were presented with a list of different AAS compounds and other IPEDs and instructed to identify what they were currently using (e.g., ancillary drugs, peptide hormones), selective androgen receptor modulators (SARMS, etc.). Items on AAS and IPED compounds were based on findings with the current literature (Brower, 2002; Hall et al., 2005; Parkinson and Evans, 2006; Westerman et al., 2016; Llewellyn et al., 2017).

#### 2.3.7 Undesired effects of AAS use

Self-report items of detrimental effects associated with AAS use were collected. Items examined the presence of physical and psychological effects currently being experienced by strength athletes who use AAS (i.e., “Are you currently experiencing any of these effects associated with the use of anabolic steroids?”). Physical effects included items well established in the current scientific literature (e.g., acne, fluid retention, injection site pain, cholesterol imbalance, elevated red blood cell count; see Amsterdam et al., 2010). Psychological effects included items associated with AAS withdrawal (e.g., depressive thoughts, decreased libido, excessive body checking, increased anxiety, insomnia, and mood swings), well established within seminal scientific literature (Brower et al., 1991; Parkinson and Evans, 2006; Ip et al., 2011; Pope et al., 2014; Westerman et al., 2016). Items were self-reported dichotomously via ‘Yes’ and ‘No’ responses.

### 2.4 Procedure

#### 2.4.1 Recruitment and data collection

Approval was granted by the authors’ institutional ethics committee (ERN_19–1955) before the study commenced. Participants were recruited through advertisements on bodybuilding and strength training forums where the use of IPEDs such as AAS is regularly discussed, social media platforms (Facebook, Twitter, Reddit), needle and syringe programs, and via existing contacts (i.e., gatekeepers). Potential participants were provided with a brief description of the study and a hyperlink to access the survey. Once accessed, participants were presented with an information sheet and consent form. Participants were informed that honesty in responding was essential to the study and that the anonymity of participants was assured as no personal details were gathered from participants. Participants were required to provide their informed consent before completing the survey, which took approximately 10–15 min. Participants were required to be individuals who use AAS had administered AAS over the last 12-months prior to the study. Data collection occurred over two phases, data from phase one (i.e., Sample 1) was analyzed before data collection began for the second phase (i.e., Sample 2). This allowed for item adjustment/creation between phases if required. Data for phase 2 was collated by the first author and a colleague from a different university with ethical approval from their institution (21/PHI/019). Upon survey completion participants were entered into a prize draw to win a £25, £50 or £100 Amazon voucher. Email addresses were collated for the purpose of the prize draw, but these were stored separately from the study data to protect anonymity.

### 2.5 Statistical analysis

Data was analyzed using IBM SPSS version 29 to conduct Pearson’s correlations, Cronbach alpha scores, and exploratory factor analysis (EFA) to determine the best indicators of each of the hypothesized factors for AAS dependence and AAS craving in Sample 1 (*n* = 206). Additionally, Mplus software version 8 (Muthén and Muthén, 2017) was used to conduct confirmatory factor analysis (CFA) to determine factorial validity in Sample 1. Mplus (Muthén and Muthén, 2017) was also used in Sample 2 (*n* = 224) to conduct CFA in order to confirm the factor structure on the proposed first-and higher-order models for AAS dependence and AAS craving (*n* = 224).

## 3 Results

### 3.1 Preliminary analyses

No missing data were present in either of the datasets. To identify the most appropriate items to measure each construct, a two-stage process recommended by Clark and Watson (2019) was used. Inter-item correlations were examined (in Sample 1 data) within each of the respective constructs and all item with correlations greater than 0.15 with all other items were retained for further analysis. EFA was then conducted within Sample 1 data for each of the nine hypothesized subscales (i.e., five dependence subscales [Effectiveness, Withdrawal, Physical, Psychological, Social] and four craving subscales [Expectation, Environment, Positive Mood, Negative Mood]) using principal axis extraction and direct oblimin rotation, with extraction based on eigenvalues ≥1.00. Subscales were analyzed individually to determine and retain the best indicators of each latent variable (Jӧreskog and Sӧrbom, 1993). Before conducting these analyses, the appropriateness of these subscales was determined by following criteria of Dziuban and Shirkey (1974) and Kaiser (1974); significant Bartlett’s test of sphericity and a Kaiser Meyer Olkin measure of sampling adequacy >0.50. All items except one had factor loadings of ≥0.52. The one exception was an AAS dependence expectation item (i.e., “I have been fearful of regressing in my training if I halted my use of steroids”), with a factor loading of 0.43, alongside a lower correlation value (*r* < 0.40) with all other items loading on that factor; this item was therefore removed from further analysis. A total of 22 items for AAS dependence (see Supplementary Table S1) and 27 items for AAS craving (see Supplementary Table S2) with minimal factor loadings of 0.50 remained for subsequent CFA.

### 3.2 Factorial validity

CFA was used to establish evidence for factorial validity due to its ability to rigorously test and confirm hypothesized factor structures (Fabrigar et al., 1999). As previously discussed, we defined AAS dependence and craving as multidimensional constructs. For dependence we expected five dimensions to be present; effectiveness, withdrawal symptoms, physical effects, psychological effects, and social effects. In turn, for craving we expected four dimensions: environment, drug expectancy, negative mood, and positive mood.

CFA analysis was conducted using Mplus software (Muthén and Muthén, 2017) using Robust Maximum Likelihood (MLR) estimation on data from Sample 1. Kolmogorov–Smirnov and Shapiro–Wilk tests indicated significant deviation from normality (*p* < 0.001), thereby requiring robust estimation. This is the default setting with Mplus and MLR estimation, producing robust standard errors, model fit indices and chi-square values (Muthén and Muthén, 2017). Multiple complimentary fit indices were used to evaluate model fit (see Hu and Bentler, 1999), specifically; Chi-square (*X^2^*), Comparative Fit Index (CFI), Standardised Root Mean Residual (SRMR), and Root Mean Square of Error Approximation (RMSEA). Good model fit is achieved when the CFI, RMSEA and SRMR values are ≥0.95, <0.06 and < 0.08, respectively, (Hu and Bentler, 1999). To compare nested models for best fit, Akaike Information Criterion (AIC) were used, with the lower value being preferred (Hair et al., 1998).

In the first AAS dependence model all 22 items were utilized and loaded onto a single factor; four items for effectiveness, nine for withdrawal, three for physical effects, three for psychological effects and three for social effects (M1a; see Table 2). Results demonstrated an inadequate model fit (Row 1), supporting the multidimensional nature of the scale and indicating a requirement for re-specification. Seven items presenting weak factor loadings and large standardized residuals were removed in a series of CFAs in which the hypothesized 5-factor model was specified. A final model with 15 items produced good model fit (M1b) with three items loading onto each of the five factors (Row 2). Each of the factors were hypothesized to represent a form of AAS dependence, therefore we thought it prudent to examine for presence of a higher order factor representing the five first-order factors. When the fit of a second-order model approaches that of a first-order model, there is sufficient support for the presence of a second-order structure (Marsh, 1993). As this was the case here (see M1c, Row 3), we accepted the higher-order model, and named the second-order factor AAS dependence.

**Table 2:** Model fit indices for each CFA model run for AAS dependence and craving measures for the first (*N* = 206) and second (*N* = 224) samples.

Model	*df*	*X^2^*	CFI	SRMR	RMSEA	AIC
**Sample 1**						
Dependence models						
1. M1a, 22-items	199	590.59	0.89	0.06	0.09	15817.64
2. M1b, 15-items	80	151.10	0.97	0.05	0.06	10570.01
3. M1c, second order 15-items	85	162.92	0.96	0.05	0.06	10571.01
Alternative dependence models						
4. M2, 15-items	90	1247.43	0.54	0.12	0.25	11646.35
5. M3, 15-items	87	853.73	0.69	0.11	0.20	11258.65
Craving models						
7. M4a, 27-items	318	909.49	0.90	0.06	0.09	17627.40
8. M4b, 16-items	98	227.44	0.96	0.04	0.08	9958.65
9. M4c, second order 16-Items	100	234.30	0.96	0.04	0.08	9961.51
Alternative craving models						
10. M5, 16-Items	104	1393.37	0.65	0.09	0.24	11262.45
11. M6, 16-items	101	598.33	0.86	0.10	0.15	10473.41
**Sample 2**						
Dependence models						
12. M1a, 15-items	80	192.16	0.96	0.04	0.07	11287.10
13. M1b, second order 15-items	85	195.01	0.96	0.04	0.07	11279.92
Craving models						
14. M2a, 16-items	98	243.07	0.97	0.03	0.08	10080.60
15. M2b, second order 16-Items	100	251.07	0.97	0.04	0.08	10084.60

df, Degrees of Freedom; X^2^, Chi-square; CFI, Comparative Fit Index; SRMR, Standardized Root Mean Square Residual; RMSEA, Root Mean Square of Error Approximation; AIC, Akaike Information Criterion. M1, five-factor model; M2, one-factor model; M3, alternate item five-factor model; M4, three-factor model; M5, three-factor model; M6, one-factor model; M7, three-factor model.

Although the data supported our hypothesized model of a five-factor structure, it was important to ensure that alternative models could be ruled out (see Table 2). We compared the model fit of the five-factor model with that of other possible structures. These were a unidimensional model with all 15 items loaded onto a single factor (M2, Row 4) and a three-factor model with undesired physical, psychological, and social effects loaded onto the same “undesired effects” factor (M3, Row 5). Table 2 demonstrates the fit of model M1b was superior to that of both alternative models. Therefore, the five-factor model was accepted as the best model for AAS dependence. Factor correlations and internal consistency scores for this model can be seen in Table 3, and items, factor loadings and error variance can be seen in Supplementary Table S3. We named the final scale the Androgenic-Anabolic Steroid Dependence Scale (AASDS).

**Table 3:** Internal consistency and correlations between factors from final AAS dependence scale models (M1c and M1b 15-item five factor models), DMDS and DSRES from Sample 1 (*N* = 206) and Sample 2 (*N* = 224).

Variable	*α*	1	2	3	4	5	6	7
1. Effectiveness	0.80/0.82	-	0.42**	0.39**	0.42**	0.41**		
2. Withdrawal	0.92/0.91	0.43**	-	0.47**	0.66**	0.59**		
3. Physical	0.94/0.95	0.31**	0.33*	-	0.53**	0.49**		
4. Psychological	0.91/0.90	0.47**	0.56**	0.43*	-	0.61**		
5. Social	0.87/0.90	0.47**	0.62**	0.32**	0.64**	-		
6. Doping MD	0.88	0.23**	0.18*	0.19**	0.29**	0.38**	-	
7. Doping SRE	0.88	−0.08	−0.12	−0.15*	−0.23**	−0.16*	−0.04	-

Sample 2 data is displayed above the diagonal. *p < 0.05; **p < 0.01; ***p < 0.001. Correlations are presented below the diagonal for Sample 1, and above the diagonal for Sample 2. Alpha scores are presented on the left-hand side for Sample 1 and on the right-hand side for Sample 2. No alpha scores are presented for doping MD and SRE for Sample 2 as these scores were only collated with Sample 1 data.

Similar procedures were followed to develop the measure for AAS craving with Sample 1 data. Initially all 27 of the items were loaded onto a single factor (M4), this model demonstrated an inadequate fit (Row 7), supporting the multidimensional nature of the scale and indicating a requirement for re-specification. Subsequently, 11 items presenting weak factor loadings and large standardized residuals were removed in a series of CFAs in which the hypothesized four-factor structure was specified. The fit of the final 16-item model with four items for each of the four factors can be seen in Table 2 (M4b), demonstrating good model fit. Each of the factors were hypothesized to represent a form of AAS craving, therefore we assessed the data for the presence of a higher order factor representing the four first-order factors. The second order model represented fit similar to that of the first order model, thus the higher-order model was accepted, and we named the second order factor AAS craving.

Alternative model structures were assessed to ensure that M4c was the best model for AAS craving. The first was with a unidimensional model with all items 16-items run on one factor (M5), indicating poor model fit. The second was a three-factor model with “positive mood” and “negative mood” combined into a single “mood” factor (M6), indicating poor fit. Table 2 contrasts the fits of the various models, demonstrating M4c as the superior model. This model was therefore accepted as the final model for AAS craving. Table 4 identifies the factor correlations and internal consistency scores, and Supplementary Table S4 indicates the items, factor loadings and error variances. We named the scale the Anabolic-Androgenic Steroid Craving Scale (AASCS).

**Table 4:** Internal consistency and correlations between factors in the final craving model (Sample 1 M4c and Sample 2 M2b 4 factor models), doping MD, doping SRE, and DSM criteria.

Variable	*α*	1	2	3	4	5	6	7
1. Expectation	0.90/0.93	-	0.62**	0.75**	0.67**			
2. Environment	0.92/0.95	0.67**	-	0.57**	0.64**			
3. Positive Mood	0.92/0.94	0.73**	0.66**	-	0.68**			
4. Negative Mood	0.98/0.98	0.61**	0.58**	0.70**	-			
5. Doping MD	0.88	0.36**	0.46**	0.45**	0.34**	-		
6. Doping SRE	0.88	−0.31**	−0.27**	−0.26**	−0.16*	−0.04	-	
7. DSM Criteria	0.82	0.55**	0.56**	0.48**	0.58**	0.27**	−0.14*	-

*p < 0.05; **p < 0.01; ***p < 0.001. Correlations are presented below the diagonal for Sample 1, and above the diagonal for Sample 2. Alpha scores are presented on the left-hand side for Sample 1 and on the right-hand side for Sample 2. No alpha scores are presented for Doping MD, Doping SRE or DSM Criteria for Sample 2 as these scores were only collated with Sample 1 data.

To further examine the construct validity of scores generated using the AASDS and AASCS, we collected evidence relating to their convergent, concurrent and discriminant validity with Sample 1. To examine convergent validity, correlations between the AAS amended DSM criteria (Kanayama et al., 2009b) with the AASDS and the AAS-WSWS (Welsch et al., 1999) with the AASCS were computed (see Table 5). Evidence for convergent validity would be established if the AASDS and AASCS were correlated at moderately high levels with the DSM criteria and AAS-WSWS, respectively. If the correlation is too high (*r* > 0.90) then the construct is redundant (Kline, 2005). There was a positive strong association between scores on the AASDS and the DSM criteria. Scores on the AASDS subscales of ‘Effectiveness’, ‘Withdrawal’, ‘Physical Effects’, ‘Psychological Effects’, and ‘Social Effects’ demonstrated moderate to strong significant positive associations with the DSM criteria. The association between AASCS scores and those from the AAS-WSWS was positive and strong. Finally, scores on the AASCS subscales of ‘, ‘Environment’, ‘Negative Mood’, and ‘Positive Mood’ were strong and positive with those from the AAS-WSWS.

**Table 5:** Descriptive statistics, internal consistencies, and correlations between AASDS, number of self-reported undesired effects, AASCS, DSM criteria, AAS-WSCS, DMDS, and DSRE from Sample 1 (*N* = 206).

	Variable	*α*	Mean	SD	Range	1	2	3	4	5	6	7
1	AASDS	0.91	3.09	1.35	1.00–7.00	-						
2	Undesired Effects		1.68	2.06	1.00–9.00	0.29**	-					
3	AASCS	0.96	3.00	1.52	1.00–7.00	0.72**	0.23**	-				
4	DSM Criteria	0.82	1.86	0.58	1.00–4.00	0.72**	0.23**	0.63**	-			
5	AAS-WSCS	0.92	2.93	1.73	1.00–7.00	0.67**	0.25**	0.85**	0.57**	-		
6	Doping MD	0.88	4.51	0.99	2.28–7.00	0.33**	0.19**	0.47**	0.27**	0.34**	-	
7	Doping SRE	0.88	3.55	1.13	1.00–5.00	−0.18**	−0.26**	−0.29**	−0.14*	−0.30**	−0.04	-

*p < 0.05; **p < 0.05; ***p < 0.001. No internal consistency was computed for Undesired effects as this was an aggregated value from a multiple-choice list of undesired effects experienced associated with AAS use, and not a measure.

Concurrent validity was assessed by measuring the associations between AASDS and experience of undesired effects (see Table 5). Past research has indicated that AAS dependence should correlate positively with experience of undesired effects associated with AAS use (Pope et al., 2010; Ip et al., 2011). Analysis demonstrated that there was a significant positive association between AASDS subscales and number of undesired effects experienced, effectiveness, withdrawal, physical effects, psychological effects, and social effects. Further evidence of concurrent validity was explored with moral disengagement (MD) and self-regulatory efficacy (SRE), as harmful use of AAS should require MD, and being dependent on AAS should be linked with low SRE. Therefore, we would expect AAS dependence to be positively associated with MD and negatively associated with SRE. These expected relationships were supported in our data, as shown in Table 5. In addition, scores on the AASDS subscales also demonstrated equivalent associations with MD and SRE (see Table 3).

Concurrent validity for AASCS scores was assessed by determining the associations of AAS craving with DMDS, DSRE and the DSM criteria scores (see Table 5). Past research has indicated that craving is associated with SRE (Shadel and Cervone, 2006), MD (Ahmadi et al., 2019), and drug dependence syndromes (Donny et al., 2008). Consistent with these previous findings, data analysis indicated significant positive associations between the AASCS subscales of expectation, environment, positive mood, and negative mood with MD (see Table 4). Further, SRE was significantly and negatively associated with expectation, environment, positive mood, and negative mood. The DSM criteria was significantly associated with expectation, environment, positive mood, and negative mood. AASCS scores demonstrated significant associations with both MD and SRE (see Table 5).

Discriminant validity of the AASDS and AASCS scores was examined through intercorrelations among subscales scores for the AASDS and AASCS. Correlations for AASDS ranged from 0.31 to 0.64 (see Table 3) indicating distinct separation between all subscale pairs. AASCS indicated correlations ranging from 0.58 to 0.73 (see Table 3), indicating distinct separation between all factors within the construct. Evidence supporting the discriminant validity of the AASCS was weaker than it was for the AASDS. However, the correlations for both measures provided support for separation of the various sub-dimensions of the AASDS and AASCS. Internal consistency of the new measures and their sub-dimensions was consistently strong (see Table 5).

### 3.3 Confirmation of factor structure

Following on from the CFA analyses on the first sample, CFA was conducted on the two (i.e., first-and higher-order) final AAS dependence models with the data gathered from Sample 2: (a) the 15-item, five-factor, first order model confirmed with data from the first sample; (b) a hierarchical model with five first-order factors and one second-order factor. CFA was also conducted on the two (i.e., first-and higher-order) final AAS craving models presented with data from the first sample: (a) the 16-item, four-factor, first order model confirmed with data from the first sample; (b) a hierarchical model with four first-order factors and one second-order factor. The results of these CFA are presented in Table 2.

The five-factor first-order AAS dependence model was the first to be evaluated (M1a). The results demonstrate a good model fit (Table 2, row 12). The second AAS dependence model analyzed was the hierarchical five-factor second-order model (M1b), presented with good model fit and achieved a similar fit to the first model (Table 2, row 13), with a very similar AIC value. Based on this we accepted M1b. Factor loadings, error variances and the final 15-items are shown in Supplementary Table S3. For AAS craving the four-factor first-order model was tested first (M2a). The model showed good model fit (Table 2, row 14). The second AAS craving model to be evaluated was the hierarchical four-factor second-order model (M2b). This model demonstrated good model fit (Table 2, row 15), with similar fit indices to the first model. Due to no major differences in the model fit we accepted M2b. Factor loadings, error variances and the final 15-items are shown in Supplementary Table S4. The final versions of the AASDS (Supplementary Table S5) and AASCS (Supplementary Table S6) can be found in the Supplementary materials.

## 4 Discussion

Research has highlighted the prevalence of AAS dependence among those who use AAS (Brower et al., 1991; Kanayama et al., 2009; Pope et al., 2014; Hauger et al., 2020; Scarth et al., 2022). It is also possible – but to date not examined – that craving plays a role in AAS dependence. However, existing measures of AAS dependence (see Kanayama et al., 2009; Griffiths et al., 2018) have significant limitations (Gossop et al., 1995; Gillespie et al., 2007; Ray et al., 2008; Kanayama et al., 2009b), and there are no validated measures to assess AAS craving. Therefore, the overarching aim of this study was to develop and validate psychometrically robust scales to assess dependence and craving within populations who use AAS.

Previous research suggested several dimensions may underlie and contribute to AAS dependence (Brower, 1992; Bahrke and Yesalis, 1994; Brower, 2002; Kanayama et al., 2010; Hildebrandt et al., 2011). Consequently, we developed a multidimensional scale and hypothesized that we would see evidence of five lower-order factors and one higher-order factor with scores obtained using the final instrument. Factor analysis of the data from both samples supported these hypotheses, providing evidence for the multifaceted nature of AAS dependence and supporting *H1a* and *H1b*. These findings are important in furthering our understanding of AAS dependence as present measures capture only a single factor (Gossop et al., 1995; Gillespie et al., 2007; Ray et al., 2008), whereas the AASDS can capture and aid our understanding of the complex multidimensional nature of AAS dependence.

The development of the AASDS as a valid and reliable multidimensional scale will further our understanding of AAS dependence via assessment of novel research questions that could not be answered with existing measures. For example, previous research has identified those dependent on AAS administer higher doses of AAS per week, and higher numbers of anabolic compounds than non-dependent individuals (see Ip et al., 2011). Now, with the AASDS, researchers may answer in-depth questions such as, ‘Which dimension of AAS dependence is linked most strongly with higher dosages of AAS?’, or ‘Which dimension of AAS dependence is associated most strongly with use of more anabolic compounds?’. In time, such research could enable harm reduction practices to tailor their approach to the specific needs of individuals with AAS dependence to deliver more effective harm reduction interventions (Bates et al., 2021).

Furthermore, the current literature indicates AAS are at times administered to self-medicate against withdrawal-like symptoms when off-cycle (Kanayama et al., 2020). Therefore, researchers could use the AASDS to address research questions such as ‘Is the subdimension of withdrawal linked with a greater propensity to shorten off-cycle periods, or progress to a blast and cruise protocol?’. Evidence also suggests a link between AAS dependence, and a greater number of self-reported undesired effects associated with use of AAS (Ip et al., 2011). The AASDS now allows researchers to investigate possible links between the three subdimensions linked to undesired effects (physiological, psychological, and social) and an increased number and severity of undesired effects. Answering questions such as these could help practitioners to identify primary areas to be targeted within therapeutic interventions and develop more effective harm reduction services for people who use AAS (Bates et al., 2019).

With no existing measure for AAS craving and evidence in the existing literature identifying the presence of craving-like behaviors in AAS research (Wood, 2008) we also sought to develop a measure of AAS craving. As existing literature indicates potential for drug craving to be multifaceted (Raabe et al., 2005; Tiffany and Wray, 2012), we aimed to develop a multidimensional scale and hypothesized the presence of four lower-order and one higher-order factor in the final instrument. Factor analysis with both samples provided support for these suppositions, therefore supporting hypotheses *H2a* and *H2b*. The development of a multidimensional measure of AAS craving is important as it will allow researchers to examine the potential role of the different dimensions of AAS craving in the development of AAS dependency specifically and use of AAS more generally.

With previous literature indicating the multi-faceted and state-like nature of craving (Shiffman et al., 1996; Sayette et al., 2000; Raabe et al., 2005), it is important to have an independent multidimensional scale to examine craving within AAS users. The four dimensions identified within this study (i.e., expectation, environment, positive and negative mood) indicate AAS craving may be experienced in a multitude of ways. Therefore, existing measures such as the DSM-V, containing a single item of craving, may not be sufficient to capture the different dimensions craving experienced within AAS users; in a reliable and valid manner (see Kline, 2000). Furthermore, as craving is considered a state-like construct (Flannery et al., 2019), it is important to separate it from trait-like phenomena such as dependence. By doing so, the AASCS will allow future research to explore the temporal patterns in craving potentially experienced by those that administer AAS.

The AASCS will allow researchers to address a wide range of important research questions that until now could not be addressed. For instance, researchers could seek to determine which dimension/s of drug craving predict increases in AAS dependence over time, whether environmentally influenced craving is associated with the amount of time athletes spend in the gym environment, or whether manipulation of athletes’ mood moderates any relationship between mood-related craving and AAS use. Addressing research questions such as these will not only benefit our current understanding of how AAS craving may contribute to AAS dependence, but it will also enable practitioners to target specific subdimensions that have stronger associations with AAS dependence and can therefore be the target of therapeutic intervention by harm reduction practitioners.

An essential component of scale development is the provision of evidence for convergent validity (Messick, 1995). Such evidence provides support that the new measure can capture the construct it was designed to assess. In terms of the study, evidence for the convergent validity of AASDS scores was demonstrated through strong positive correlations with the AAS adapted DSM criteria (Kanayama et al., 2009b) and of AASCS scores through associations with the AAS adapted WSWS (Welsch et al., 1999). These associations are consistent with recommendations for establishing convergent validity (Kline, 2005; Byrne, 2012). This is important as it provides evidence that the AASDS and AASCS assess their respective psychological constructs of dependence and craving. In terms of the AASDS, this is particularly important given evidence for the convergent validity of the DSM-V is presently absent (Welch et al., 2013).

Another crucial element of establishing construct validity is providing evidence for concurrent validity (Kline, 2005). Here, evidence should be provided showing the new scale can predict theoretically related constructs when data on the two constructs are collected at the same time (Kline, 2005). Presently, we provided evidence for the concurrent validity of AASDS scores by exhibiting strong positive correlations between AASDS scores and self-reported undesired effects from AAS use, whereas for AASCS scores we demonstrated a moderate positive correlation with doping MD and a moderate negative association with doping SRE (Boardley et al., 2018). Support for the concurrent validity of AASDS and AASCS scores is important, as it indicates scores with both measures can predict theoretically related constructs.

Correlations examining concurrent validity showed differences across the subscales of the AASDS and AASCS. For the AASDS, the “*use of AAS despite experiencing associated undesired physical effects*” subscale had the strongest association with self-reported experience of undesired effects, and “*use of AAS despite experiencing associated undesired social effects*” the weakest. This is consistent the extant literature on AAS dependence, whereby AAS dependent users reported experiencing more undesired physical effects while using AAS (Ip et al., 2011), and reported a higher frequency of undesired physical effects compared to undesired psychological and social effects (Pope et al., 2010; Ip et al., 2011). For the AASCS, the “*environmental cues*” and “*AAS expectancy*” subscales had the strongest associations with doping MD and doping SRE, and “*negative mood*” indicated the weakest association with these constructs. These findings are consistent with non-AAS research on craving, where SRE has shown a negative association with craving (Shadel and Cervone, 2006) and a positive association with MD (Ahmadi et al., 2019). The differential associations with theoretically related constructs for the AASDS and AASCS subscales are important as they provide evidence the subscales are capturing different elements of dependency and craving.

Evidence for discriminant validity is a key consideration when validating new scales (Messick, 1995). Moderate evidence of discriminant validity was established for the AASDS as subscale scores were moderately to strongly intercorrelated, indicating while they are all underpinned by a higher-order dependency construct, they are distinct from one another. For AASCS scores, there was evidence of discriminant validity through strong intercorrelations between subscales, evidencing each subscale was underpinned by a higher-order craving construct while being distinct from each other. By providing evidence of discriminant validity, we have demonstrated the items within the respective subscales are related but distinct from one another (Clark and Watson, 2019).

Finally, new measures should also provide evidence supporting the internal consistency of scale and subscale scores as this provides evidence for the homogeneity of items (Clark and Watson, 2019). Assessment of the internal consistency values for both the AASDS and AASCS and their respective subscales exceeded the minimum criterion levels recommended when developing novel scales (i.e., 0.80; Clark and Watson, 2019) in both samples. This provides strong and consistent evidence for the internal consistency of scale scores for the AASDS and AASCS, as well as their respective subscales.

The development and validation of the AASDS and AASCS provides several advantages to researchers. Previous scales used to measure AAS dependence, such as the DSM-IV criteria, required administration by trained clinicians (Pope et al., 2010). The development of the AASDS now means researchers can assess AAS dependence without the need for a trained clinician, therefore making its assessment easier and more accessible. Also, to date research on AAS dependence has largely assessed it as a binary construct (i.e., it is present or it is not; see Kanayama et al., 2009a). However, researchers have recently contradicted this and have argued that AAS dependence is a continuous construct (see Scarth et al., 2022). The development of the AASDS is therefore timely, as it represents the first validated measure of AAS dependence that assesses it as a continuous construct. Finally, the latest edition of the DSM criteria includes an item on craving in its classification of substance use disorder. While there is support for craving as a criterion for diagnosis of substance dependence, currently there is no consensus on whether craving merely cooccurs with dependence or is actually a sub-component of it (Tiffany and Wray, 2012). Our development of separate measures of AAS dependence and AAS craving provides researchers with distinct measures of the two constructs that will facilitate research to help elucidate our understanding on this.

### 4.1 Limitations and future directions

This research developed and validated the scores of two multidimensional psychological instruments measuring AAS dependence and craving, providing convincing evidence for the psychometric properties of both scales. However, this research was not without limitations which should be acknowledged. First, it is possible that individuals recruited within the study may not reflect all types of AAS user. To elaborate, Christiansen et al. (2017) developed a typology of AAS user that described four types of AAS user. One of the four types was termed the ‘You only live once’ (YOLO) type. The YOLO type is characterized by primarily obtaining information on AAS from steroid gurus and often being distrustful of medical professionals and/or researchers (Bonnecaze et al., 2020). As a result of this lack of trust, it can be difficult to recruit this particular group within research with people who use AAS. As such, this sub-population may have been underrepresented in our sample, and there may be a need for future research that specifically targets members of the YOLO type to ensure the validity of scores obtained with the AASDS and AASCS with this group. Additionally, symptoms of dependence and craving may have varied depending on the duration of AAS use and how recently AAS had been administered. Therefore, future studies may wish to assess symptoms of AAS dependence and craving in different populations of AAS users (i.e., long-term users compared to short-term users, and in different statuses of use such as on-cycle, off-cycle, blasting, and cruising).

Another limitation of our research was that the majority (>90%) of our participants were male. As a result, we were not able to assess whether the factor structure of the two scales was invariant between males and females or examine other elements of validity and reliability specifically with females. Sensitivity analyses, with female participants removed, revealed similar model fit and factor loadings to the analyses with female participants included (see Supplementary Tables S7–S9). It would be interesting in future research to validate AASDS and AASCS scores female only samples of AAS users. To aid in this recruitment process it will be imperative to identify key female gatekeepers within the online fora, as this sub-group of AAS users is hard to reach and as a result remains understudied (Henning and Andreasson, 2019). Furthermore, not all our participants were from native English-speaking nations. Sample 1 and Sample 2 consisted of 88 and 90% native English-speaking participants respectively, which may have impacted the interpretation of items and negatively influenced the factor structure of the scales. Future studies would be advised to conduct a validation of these measures with native English-speaking participants only. Despite the differences identified between sample populations (see Table 1), there may have been a small degree of overlap between the two samples due to the online nature of data collection. Ideally the two samples should be entirely independent, and due to the methods of data collection we cannot be certain this is the case. Future validation studies should seek to ensure entirely independent samples are recruited.

A further limitation is that we did not examine all aspects of validity. Validation of measures is an ongoing process (Clark and Watson, 2019), and researchers are encouraged to examine other aspects of validity of AASDS and AASCS scores in future work. For instance, researchers could examine the predictive validity of scores through longitudinal research that examines possible links with theoretically related constructs over time. For instance, associations between AASDS and AASCS scores and emotional states (e.g., positive and negative affect) could be assessed. In addition, the data for our study was collected from gym users. While it is likely a sizeable proportion of our participants played sport alongside their gym activities; we did not collect data from sport participants who do not attend gyms. Thus, it is important in future work to validate AASDS and AASCS scores with sport participants who do not frequent gyms. Our data were collected largely from westernized cultures, too. Further validation of the scales with non-westernized cultures is therefore another important avenue for future work. Finally, it was beyond the scope of this article to measure test–retest reliability to establish the consistency of AASDS and AASCS scores across time, and as such future researchers should aim to address this. For those engaging in such work, we recommend brief time intervals between data collection periods for craving, given it is a state-like construct (Shiffman et al., 1996; Sayette et al., 2000). In contrast, longer time intervals could be used for dependence given it is a more enduring construct (see American Psychiatric Association DSM-5, Task Force, 2013). It is important to keep this in mind, as lower stability scores from craving may reflect changes in levels of craving over time rather than inconsistencies in measurement (Sayette et al., 2000).

## 5 Conclusion

Through a rigorous set of procedures, we developed psychometric scales to assess AAS dependence and AAS craving, providing convincing evidence for the validity of scores obtained with both measures. Specifically, evidence for several aspects of construct validity (i.e., convergent, concurrent and discriminant validity) and internal consistency was provided for the AASDS and AASCS. During item development we also provided evidence for high levels of content and face validity of items through feedback from relevant experts. The AASDS makes important contribution to the literature as it is the first AAS dependency measure specifically designed for those who use AAS rather than being adapted from existing measures. In turn, the development of the AASCS is important because it represents the first AAS craving measure. Further, the multidimensional nature of both measures provides exciting possibilities for future research. We look forward to seeing further evaluation and use of these measures in future research.

## Data availability statement

The raw data supporting the conclusions of this article will be made available by the authors, without undue reservation.

## Ethics statement

The studies involving humans were approved by University of Birmingham Ethics Committee (ERN_19-1955). The studies were conducted in accordance with the local legislation and institutional requirements. The participants provided their written informed consent to participate in this study.

## Author contributions

BZ: Writing – original draft, Writing – review & editing. IB: Writing – original draft, Writing – review & editing.

## Footnotes

^1^Misuse is used here to refer to the use of a substance in a manner that is not medically recommended (World Health Organization, 1992).

^2^There have been no reported cases of AAS dependence whilst using therapeutic doses of AAS (Brower, 2002).

^3^The term ‘dependence’ is utilised throughout this study as this terminology is widely accepted within the AAS literature.

^4^The majority of extant research on AAS dependence utilises the DSM-IV criteria (Kanayama et al., 2010; which does not include craving) rather than the DSM-V (which includes craving; American Psychiatric Association DSM-5, Task Force, 2013).

^5^Kanayama et al. (2010) theoretical three-mechanism model of AAS dependence was used in the creation of items for the AAS dependence item pool. Niaura’s (2000) theory of drug craving was used to create items for the AAS craving item pool.

^6^38 academics and healthcare workers were contacted to be considered members of the expert panel; 22 replied positively and took part in the study.

^7^Questionnaires differed in Sample 1 and Sample 2. Data collected from Sample 1 included measures of AAS dependence (DSM criteria), Moral Disengagement (DMDS), Self-Regulatory Efficacy (DSRE), the AAS adapted Wisconsin Smoking Withdrawal Scale (AAS-WSWS), items assessing patterns of use of AAS, and self-reported items on the experience of undesired effects associated with the use of AAS. Whilst data collated from Sample 2 only included the new measures as it was intended to test for factorial validity alone.
