# Supplementary material for: Development and validation of dependence and craving measures specific to athletes who use anabolic-androgenic steroids
Source: Front Psychol. 2024 Aug 8;15:1347211. doi: 10.3389/fpsyg.2024.1347211 (PMC11339692; doi:10.3389/fpsyg.2024.1347211)
Supplement: Supplementary file 1 [file Data_Sheet_1.pdf]

Supplementary Materials

Appendix 1

Table S1. Exploratory factor analysis standardised factor loadings and error variances for the AAS dependence scale (AASDS) from Sample 1 (*N* = 206)

Factor	Factor Loadings	Error Variances
Item		
Effectiveness		
I have increased the overall amount of steroids I am using to make my regime more effective.	0.50	0.75
I have increased my use of steroids due to dissatisfaction with the effectiveness of my regime.	0.67	0.56
I have gone beyond my pre-planned use of steroids to increase my gains.	0.85	0.29
I have increased my use of steroids to increase gains.	0.80	0.36
Withdrawal		
I have continued to use steroids because I anticipate unwanted effects if I stop.	0.50	0.75
I have found it difficult to go without steroids due to a loss of gains when off-cycle.	0.58	0.66
I have shortened a pre-planned steroid "off-cycle" period to avoid withdrawal symptoms.	0.78	0.39
I have lengthened a pre-planned steroid "on-cycle" period to avoid withdrawal symptoms.	0.74	0.46
I have restarted using steroids because I experienced unwanted effects during an off-cycle period.	0.81	0.35
I have used steroids to alleviate effects induced by stopping my use.	0.81	0.35
Experiencing withdrawal symptoms has made it difficult to stop using steroids during “off-cycle” periods.	0.92	0.16
I have used steroids to alleviate withdrawal symptoms experienced during an “off-cycle” period.	0.82	0.33
I had a strong compulsion to use steroids when “off-cycle” due to experiencing withdrawal-like symptoms.	0.84	0.30
Physical		
I have continued using steroids despite experiencing unwanted side effects (e.g., gynecomastia, heart complications, cholesterol imbalance, abscesses, tendon/joint damage, testicular atrophy).	0.93	0.13
I have continued to use steroids despite trying to manage undesired side effects (e.g., such as; gynecomastia, heart complications, cholesterol imbalance, abscesses from injections, tendon/joint damage, testicular atrophy).	0.90	0.20
I have continued with my steroid regime since experiencing unwanted effects (e.g., such as; gynecomastia, heart complications, cholesterol imbalance, abscesses from injections, tendon/joint damage, testicular atrophy).	0.93	0.13
Psychological		

I have continued with my steroid regime despite seeking help for problematic psychological effects (e.g., depressive thoughts, a decreased libido, increased anxiety, insomnia, and mood swings).	0.85	0.27
Experiencing unwanted side effects (e.g., depressive thoughts, decreased libido, increased anxiety, insomnia, mood swings) has concerned me, but I continue to use steroids.	0.91	0.18
I have experienced depressive thoughts, a decreased libido, increased anxiety, insomnia and mood swings, and continued using steroids.	0.89	0.21
Social		
I have avoided social, occupational and/or recreational activities as they would have interfered with my steroid regime.	0.67	0.56
I always prioritise my steroid regime over social, occupational and/or recreational activities, even if the outcome may be problematic.	0.93	0.14
Avoiding social, occupational and/or recreational activities to prioritise my steroid regime has caused me problems within my personal life (i.e., with close family, friends, partner/significant other, boss/manager).	0.94	0.13

Appendix 2

Table S2. Exploratory factor analysis standardised factor loadings and error variances for the AAS craving scale (AASCS) from Sample 1 (*N* = 206)

Factor	Factor Loadings	Error Variances
Item		
Expectation		
I have trouble getting steroids off my mind because of what they can do for me.	0.88	0.22
I frequently think about my steroid routine because of how it makes me feel.	0.89	0.21
Much of my time is occupied by ideas, thoughts, impulses, and images relating to what I can achieve whilst using steroids.	0.87	0.24
It takes a lot of effort to disregard my thoughts and feelings about my use of steroids.	0.86	0.26
I frequently think about how being on steroids makes me feel	0.83	0.31
I have reoccurring thoughts about how using steroids will help me reach my goals.	0.74	0.45
I have an uncontrollable desire to use steroids.	0.72	0.48
I have strong urges to increase my steroid use when not performing well.	0.74	0.45
I have a strong need to use steroids due to knowing how they can enhance my progress.	0.76	0.43
Environment		
Talking to other gym users about training makes me want to use steroids.	0.81	0.35
Going to the gym makes me desire the use of steroids.	0.75	0.44
Being around my gym friends makes me want to use steroids.	0.94	0.13
Being around my gym friends makes me desire steroids.	0.90	0.20
My desire to use steroids when surrounded by my gym friends is overwhelming.	0.73	0.46
Just passing by a gym can make me want to use steroids.	0.94	0.12
Positive Mood		
I only anticipate positive effects associate with taking steroids.	0.69	0.52
I get excited at the thought of using steroids.	0.70	0.51
The thought of using steroids makes me feel more relaxed.	0.71	0.50
I feel content when anticipating using steroids.	0.83	0.31
The thought of using steroids improves my mood.	0.83	0.32
Knowing I will be using steroids improves my mood.	0.93	0.13
Negative Mood		
I have a desire to use steroids when I am feeling down.	0.95	0.09
I desire to use steroids when I feel irritable.	0.97	0.06
The feeling of being down makes me desire steroids.	0.96	0.09
I have an urge to use steroids when I feel anxious.	0.94	0.13
I have a compulsion to use steroids when feeling tense.	0.98	0.02

Appendix 3

Table S3. M1c Items, standardized factor loadings and error variances for the AAS dependence scale (AASDS) from Sample 1 (*N* = 206) and Sample 2 (*N* = 224).

Factor	Factor Loadings	Error Variances
Item		
Effectiveness		
1. I have increased my use of steroids due to dissatisfaction with the effectiveness of my regime.	0.63/0.68	0.60/0.53
2. I have gone beyond my pre-planned use of steroids to increase my gains.	0.88/0.85	0.22/0.26
3. I have increased my use of steroids to increase gains.	0.78/0.81	0.38/0.33
Withdrawal		
4. I have used steroids to alleviate effects induced by stopping my use.	0.81/0.80	0.34/0.34
5. I have used steroids to alleviate withdrawal symptoms experienced during an “off-cycle” period.	0.93/0.93	0.12/0.12
6. Experiencing withdrawal symptoms has made it difficult to stop using steroids during “off-cycle” periods.	0.94/0.92	0.11/0.13
Unwanted Physical Effects		
7. I have continued using steroids despite experiencing unwanted side effects (e.g., gynecomastia, heart complications, cholesterol imbalance, abscesses, tendon/joint damage, testicular atrophy).	0.93/0.91	0.12/0.17
8. I have continued to use steroids despite trying to manage undesired side effects (e.g., such as; gynecomastia, heart complications, cholesterol imbalance, abscesses from injections, tendon/joint damage, testicular atrophy).	0.89/0.96	0.20/0.07
9. I have continued with my steroid regime since experiencing unwanted effects (e.g., such as; gynecomastia, heart complications, cholesterol imbalance, abscesses from injections, tendon/joint damage, testicular atrophy).	0.93/0.91	0.13/0.16
Unwanted Psychological Effects		

10. I have continued with my steroid regime despite seeking help for problematic psychological effects (e.g., depressive thoughts, a decreased libido, increased anxiety, insomnia, and mood swings).	0.87/0.81	0.23/0.34
11. I have experienced depressive thoughts, a decreased libido, increased anxiety, insomnia and mood swings, and continued using steroids.	0.88/0.93	0.21/0.13
12. Experiencing unwanted side effects (e.g., depressive thoughts, decreased libido, increased anxiety, insomnia, mood swings) has concerned me, but I continue to use steroids.	0.89/0.87	0.20/0.23
Unwanted Social Effects		
13. I have avoided social, occupational and/or recreational activities as they would have interfered with my steroid regime.	0.67/0.81	0.55/0.34
14. Avoiding social, occupational and/or recreational activities to prioritise my steroid regime has caused me problems within my personal life (i.e., with close family, friends, partner/significant other, boss/manager).	0.94/0.90	0.10/0.18
15. I always prioritise my steroid regime over social, occupational and/or recreational activities, even if the outcome may be problematic.	0.91/0.91	0.16/0.15

Appendix 4

Table S4. M4c Items, standardized factor loadings and error variances for the AAS craving scale (AASCS) from Sample 1 (*N* = 206) and Sample 2 (*N* = 224).

Factor	Factor Loadings	Error Variances
Item		
Expectation		
1. I have trouble getting steroids off my mind because of what they can do for me.	0.84/0.91	0.29/0.16
2. I frequently think about my steroid routine because of how it makes me feel.	0.82/0.89	0.31/0.20
3. Much of my time is occupied by ideas, thoughts, impulses, and images relating to what I can achieve whilst using steroids.	0.85/0.87	0.28/0.23
4. It takes a lot of effort to disregard my thoughts and feelings about my use of steroids.	0.80/0.87	0.35/0.24
Environment		
5. Being around my gym friends makes me want to use steroids.	0.91/0.93	0.15/0.12
6. Talking to other gym users about training makes me want to use steroids.	0.90/0.93	0.18/0.13
7. Being around my gym friends makes me desire steroids.	0.74/0.91	0.45/0.16
8. Just passing by a gym can make me want to use steroids.	0.94/0.89	0.10/0.19
Positive Mood		
9. The thought of using steroids makes me feel more relaxed.	0.82/0.86	0.32/0.25
10. The thought of using steroids improves my mood.	0.82/0.89	0.32/0.20
11. Knowing I will be using steroids improves my mood.	0.93/0.92	0.12/0.14
12. I feel content when anticipating using steroids.	0.90/0.93	0.19/0.12
Negative Mood		
13. I have a desire to use steroids when I am feeling down.	0.95/0.90	0.08/0.17
14. I desire to use steroids when I feel irritable.	0.96/0.98	0.06/0.02

15. I have an urge to use steroids when I feel anxious.	0.97/0.98	0.05/0.02
16. I have a compulsion to use steroids when feeling tense.	0.95/0.98	0.09/0.02

Appendix 5

Table S5. *The Anabolic-Androgenic Steroid Dependence Scale*

A number of statements describing experiences and scenarios you may have had whilst using anabolic steroids are presented below, please rate your level of agreement with the following items.

Over the last 12-months,	Strongly Disagree	Disagree	Slightly Disagree	Neutral	Slightly Agree	Agree	Strongly Agree
1. I have increased my use of steroids due to dissatisfaction with the effectiveness of my regime.	1	2	3	4	5	6	7
2. I have gone beyond my pre-planned use of steroids to increase my gains.	1	2	3	4	5	6	7
3. I have increased my use of steroids to increase gains.	1	2	3	4	5	6	7
4. I have used steroids to alleviate effects induced by stopping my use.	1	2	3	4	5	6	7
5. I have used steroids to alleviate withdrawal symptoms experienced during an “off-cycle” period.	1	2	3	4	5	6	7
6. Experiencing withdrawal symptoms has made it difficult to stop using steroids during “off-cycle” periods.	1	2	3	4	5	6	7
7. I have continued using steroids despite experiencing unwanted side effects (e.g., gynecomastia, heart complications, cholesterol imbalance, abscesses, tendon/joint damage, testicular atrophy).	1	2	3	4	5	6	7
8. I have continued to use steroids despite trying to manage undesired side effects (e.g., such as; gynecomastia, heart complications, cholesterol imbalance, abscesses from injections, tendon/joint damage, testicular atrophy).	1	2	3	4	5	6	7

9. I have continued with my steroid regime since experiencing unwanted effects (e.g., such as; gynecomastia, heart complications, cholesterol imbalance, abscesses from injections, tendon/joint damage, testicular atrophy).	1	2	3	4	5	6	7
10. I have continued with my steroid regime despite seeking help for problematic psychological effects (e.g., depressive thoughts, a decreased libido, increased anxiety, insomnia, and mood swings).	1	2	3	4	5	6	7
11. I have experienced depressive thoughts, a decreased libido, increased anxiety, insomnia and mood swings, and continued using steroids.	1	2	3	4	5	6	7
12. Experiencing unwanted side effects (e.g., depressive thoughts, decreased libido, increased anxiety, insomnia, mood swings) has concerned me, but I continue to use steroids.	1	2	3	4	5	6	7
13. I have avoided social, occupational and/or recreational activities as they would have interfered with my steroid regime.	1	2	3	4	5	6	7
14. Avoiding social, occupational and/or recreational activities to prioritise my steroid regime has caused me problems within my personal life (i.e., with close family, friends, partner/significant other, boss/manager).	1	2	3	4	5	6	7
15. I always prioritise my steroid regime over social, occupational and/or recreational activities, even if the outcome may be problematic.	1	2	3	4	5	6	7

Appendix 6

Table S6. *The Anabolic-Androgenic Steroid Craving Scale*

A number of statements describing thought and experiences you may have had whilst using anabolic steroids are presented below, please rate your level of agreement with the following items.

Presently,	Strongly Disagree	Disagree	Slightly Disagree	Neutral	Slightly Agree	Agree	Strongly Agree
1. I have trouble getting steroids off my mind because of what they can do for me.	1	2	3	4	5	6	7
2. I frequently think about my steroid routine because of how it makes me feel.	1	2	3	4	5	6	7
3. Much of my time is occupied by ideas, thoughts, impulses, and images relating to what I can achieve whilst using steroids.	1	2	3	4	5	6	7
4. It takes a lot of effort to disregard my thoughts and feelings about my use of steroids.	1	2	3	4	5	6	7
5. Being around my gym friends makes me want to use steroids.	1	2	3	4	5	6	7
6. Talking to other gym users about training makes me want to use steroids.	1	2	3	4	5	6	7
7. Being around my gym friends makes me desire steroids.	1	2	3	4	5	6	7
8. Just passing by a gym can make me want to use steroids.	1	2	3	4	5	6	7
9. The thought of using steroids makes me feel more relaxed.	1	2	3	4	5	6	7
10. The thought of using steroids improves my mood.	1	2	3	4	5	6	7
11. Knowing I will be using steroids improves my mood.	1	2	3	4	5	6	7
12. I feel content when anticipating using steroids.	1	2	3	4	5	6	7

13. I have a desire to use steroids when I am feeling down.	1	2	3	4	5	6	7
14. I desire to use steroids when I feel irritable.	1	2	3	4	5	6	7
15. I have an urge to use steroids when I feel anxious.	1	2	3	4	5	6	7
16. I have a compulsion to use steroids when feeling tense.	1	2	3	4	5	6	7

Appendix 7

Table S7. Model fit indices for each CFA model run for AAS dependence and craving measures for the first (*N* = 206) and second (*N* =224) samples.

Model	*df*	X^2	CFI	SRMR	RMSEA	AIC
			**Sample 1**			
**Dependence Models**						
1. M1a, 22-Items	199	590.59	0.89	0.06	0.09	15817.64
2. M1b, 15-Items	80	151.10	0.97	0.05	0.06	10570.01
3. M1c, Second Order 15-Items	85	162.92	0.96	0.05	0.06	10571.01
4. M1c Second Order 15-Items (NF)	85	175.20	0.96	0.05	0.06	10511.00
**Alternative Dependence Models**						
5. M2, 15-Items	90	1247.43	0.54	0.12	0.25	11646.35
6. M3, 15-Items	87	853.73	0.69	0.11	0.20	11258.65
**Craving Models**						
8. M4a, 27-Items	318	909.49	0.90	0.06	0.09	17627.40
9. M4b, 16-Items	98	227.44	0.96	0.04	0.08	9958.65
10. M4c, Second Order 16-Items	100	234.30	0.96	0.04	0.08	9961.51
11. M4c, Second Order 16-Items (NF)	100	239.79	0.97	0.05	0.08	9934.31
**Alternative Craving Models**						
12. M5, 16-Items	104	1393.37	0.65	0.09	0.24	11262.45
13. M6, 16-Items	101	598.33	0.86	0.10	0.15	10473.41
			**Sample 2**			
**Dependence Models**						
14. M1a, 15-Items	80	192.16	0.96	0.04	0.07	11287.10
15. M1b, Second Order 15-Items	85	195.01	0.96	0.04	0.07	11279.92
16. M1b, Second Order 15-Items (NF)	85	190.99	0.96	0.04	0.07	11045.82
**Craving Models**						
17. M2a, 16-Items	98	243.07	0.97	0.03	0.08	10080.60
18. M2b, Second Order 16-Items	100	251.07	0.97	0.04	0.08	10084.60
19. M2b, Second Order 16-Items (NF)	100	247.59	0.97	0.04	0.08	10816.92

Note: df = Degrees of Freedom, X^2 = Chi-square, CFI = Comparative Fit Index, SRMR = Standardized Root Mean Square Residual, RMSEA = Root Mean Square of Error Approximation, AIC = Akaike Information Criterion. M1 = five-factor model; M2 = one-factor model; M3 = alternate item five-factor model; M4 = three-factor model; M5 = three-factor model; M6 = one-factor model; M7 = three-factor model, NF = No females for sensitivity analysis in Sample 1 (*n* = 20) and in Sample 2 (*n* = 8).

Appendix 8

Table S8. M1c Items, standardized factor loadings and error variances for the AAS dependence scale (AASDS) from male participants only in Sample 1 (*N* = 186) and Sample 2 (*N* = 216).

Factor<br>Item	Factor Loadings	Error Variances
Effectiveness		
1. I have increased my use of steroids due to dissatisfaction with the effectiveness of my regime.	0.65/0.68	0.58/0.53
2. I have gone beyond my pre-planned use of steroids to increase my gains.	0.88/0.86	0.23/0.27
3. I have increased my use of steroids to increase gains.	0.80/0.81	0.36/0.34
Withdrawal		
4. I have used steroids to alleviate effects induced by stopping my use.	0.82/0.80	0.33/0.36
5. I have used steroids to alleviate withdrawal symptoms experienced during an “off-cycle” period.	0.93/0.94	0.13/0.12
6. Experiencing withdrawal symptoms has made it difficult to stop using steroids during “off-cycle” periods.	0.94/0.93	0.11/0.14
Unwanted Physical Effects		
7. I have continued using steroids despite experiencing unwanted side effects (e.g., gynecomastia, heart complications, cholesterol imbalance, abscesses, tendon/joint damage, testicular atrophy).	0.94/0.91	0.12/0.17
8. I have continued to use steroids despite trying to manage undesired side effects (e.g., such as; gynecomastia, heart complications, cholesterol imbalance, abscesses from injections, tendon/joint damage, testicular atrophy).	0.90/0.93	0.20/0.07
9. I have continued with my steroid regime since experiencing unwanted effects (e.g., such as; gynecomastia, heart complications, cholesterol imbalance, abscesses from injections, tendon/joint damage, testicular atrophy).	0.93/0.87	0.13/0.16
Unwanted Psychological Effects		
10. I have continued with my steroid regime despite seeking help for problematic psychological effects (e.g., depressive thoughts, a decreased libido, increased anxiety, insomnia, and mood swings).	0.88/0.81	0.23/0.35

11. I have experienced depressive thoughts, a decreased libido, increased anxiety, insomnia and mood swings, and continued using steroids.	0.88/0.90	0.22/0.14
12. Experiencing unwanted side effects (e.g., depressive thoughts, decreased libido, increased anxiety, insomnia, mood swings) has concerned me, but I continue to use steroids.	0.88/0.92	0.20/0.24
Unwanted Social Effects		
13. I have avoided social, occupational and/or recreational activities as they would have interfered with my steroid regime.	0.66/0.81	0.56/0.34
14. Avoiding social, occupational and/or recreational activities to prioritise my steroid regime has caused me problems within my personal life (i.e., with close family, friends, partner/significant other, boss/manager).	0.95/0.90	0.10/0.19
15. I always prioritise my steroid regime over social, occupational and/or recreational activities, even if the outcome may be problematic.	0.93/0.91	0.14/0.16

Appendix 9

Table S9. M4C Items, standardized factor loadings and error variances for the AAS craving scale (AASCS) from male participants only in Sample 1 (*N* = 186) and Sample 2 (*N* = 216).

Factor<br>Item	Factor Loadings	Error Variances
Expectation		
1. I have trouble getting steroids off my mind because of what they can do for me.	0.85/0.91	0.28/0.17
2. I frequently think about my steroid routine because of how it makes me feel.	0.82/0.90	0.32/0.20
3. Much of my time is occupied by ideas, thoughts, impulses, and images relating to what I can achieve whilst using steroids.	0.85/0.87	0.28/0.24
4. It takes a lot of effort to disregard my thoughts and feelings about my use of steroids.	0.81/0.87	0.35/0.24
Environment		
5. Being around my gym friends makes me want to use steroids.	0.92/0.94	0.16/0.12
6. Talking to other gym users about training makes me want to use steroids.	0.91/0.93	0.18/0.13
7. Being around my gym friends makes me desire steroids.	0.73/0.91	0.46/0.17
8. Just passing by a gym can make me want to use steroids.	0.94/0.89	0.11/0.20
Positive Mood		
9. The thought of using steroids makes me feel more relaxed.	0.82/0.86	0.32/0.25
10. The thought of using steroids improves my mood.	0.81/0.89	0.34/0.20
11. Knowing I will be using steroids improves my mood.	0.94/0.92	0.12/0.15
12. I feel content when anticipating using steroids.	0.90/0.93	0.20/0.13
Negative Mood		
13. I have a desire to use steroids when I am feeling down.	0.96/0.87	0.09/0.17
14. I desire to use steroids when I feel irritable.	0.97/0.89	0.06/0.02
15. I have an urge to use steroids when I feel anxious.	0.97/0.93	0.05/0.02

16. I have a compulsion to use steroids when feeling tense.	0.95/0.94	0.10/0.03
